# Silent Superior Mesenteric Artery Dissection Discovered During Hypertensive Emergency With Acute Pulmonary Edema in an End-Stage Renal Disease Patient

**DOI:** 10.7759/cureus.106707

**Published:** 2026-04-09

**Authors:** Tutul Chowdhury, Nisha K Sapkota, Aditi Parulkar, Anusha Akella, Mehak G Mastoi, Minhaz Murshad, Mujibur Majumder

**Affiliations:** 1 Pulmonary and Critical Care Medicine, Interfaith Medical Center, Brooklyn, USA; 2 Pulmonary and Critical Care Medicine, Brookdale University Hospital Medical Center, Brooklyn, USA; 3 Medicine, Interfaith Medical Center, Brooklyn, USA; 4 Internal Medicine, One Brooklyn Health, Brooklyn, USA; 5 Internal Medicine, Interfaith Medical Center, Brooklyn, USA; 6 Geriatrics, Montefiore Medical Center - Wakefield Campus, Brooklyn, USA; 7 Internal Medicine, Jersey Shore University Medical Center, Neptune, USA

**Keywords:** acute pulmonary edema, biphasic t-wave, end-stage renal disease, hypertensive emergency, intimal injury, superior mesenteric artery dissection

## Abstract

Isolated superior mesenteric artery (SMA) dissection is a rare vascular condition that is increasingly recognized due to the widespread use of advanced imaging modalities. It may present with abdominal pain or remain asymptomatic and be detected incidentally. We report the case of a 62-year-old male with end-stage renal disease (ESRD) on hemodialysis who presented with hypertensive emergency and acute pulmonary edema, in whom SMA dissection was incidentally identified on computed tomography angiography (CTA). The patient was successfully managed conservatively with blood pressure control, antithrombotic therapy, and close monitoring. This case highlights the importance of individualized management strategies in asymptomatic SMA dissection and supports the role of conservative therapy in stable patients without evidence of bowel ischemia.

## Introduction

Isolated spontaneous dissection of the superior mesenteric artery (ISMAD) is an uncommon but increasingly recognized vascular pathology, largely due to advances in imaging modalities, such as computed tomography angiography (CTA) [1,2]. Clinical presentation is highly variable, ranging from acute abdominal pain to entirely asymptomatic cases detected incidentally [3,4]. Hypertension and atherosclerosis are among the most frequently associated risk factors, likely contributing to increased arterial wall stress and intimal injury [5,6]. Management remains controversial and may include conservative therapy, antithrombotic treatment, endovascular intervention, or surgical repair, depending on clinical presentation and the presence of complications, such as bowel ischemia [3,7]. We report a case of asymptomatic superior mesenteric artery (SMA) dissection identified during evaluation of hypertensive emergency and acute pulmonary edema in a patient with end-stage renal disease (ESRD).

## Case presentation

**Figure 1:**
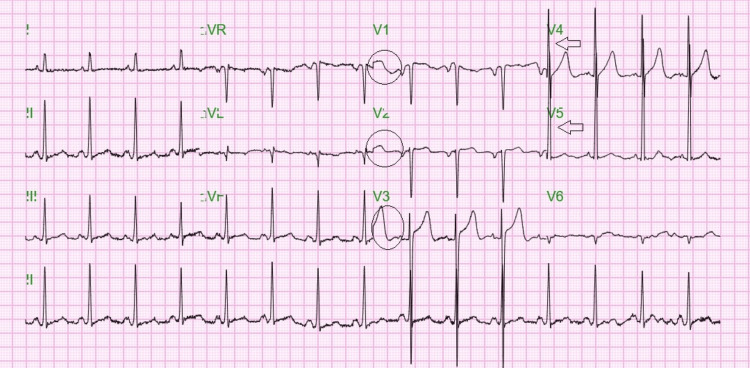
Electrocardiogram showing signs of demand ischemia with biphasic T wave in V1, V2, and V3 leads (black circles) and left ventricular hypertrophy depicted as R wave >26 millimeters in lead V4 and V5 (black arrows).

A 62-year-old man with chronic kidney disease on thrice-weekly hemodialysis, hypertension, and hyperlipidemia presented following a hemodialysis session with progressively worsening shortness of breath and new-onset lower extremity edema. On arrival, his blood pressure was 206/138 mmHg. Physical examination revealed jugular venous distension and diffuse crackles on lung auscultation, while cardiac examination was unremarkable. The abdomen was soft and non-tender, without signs of peritonitis. The patient denied chest pain and had no acute gastrointestinal symptoms. Laboratory evaluation revealed elevated high-sensitivity troponin levels without ST elevation on electrocardiogram (Figure 1), consistent with demand ischemia, and a brain natriuretic peptide (BNP) level >2,000 pg/mL.

There was no evidence of lactic acidosis. Hemoglobin was approximately 10 g/dL, consistent with chronic normocytic anemia, and the platelet count was 90,000/µL, presumed to be reactive in the setting of acute illness. Labs on admission are categorized in Table 1.

**Table 1:** Labs on admission. PCR: Polymerase chain reaction

Investigation	Value, Day 1	Value, Day 7	Reference range
Hemoglobin	9.4	8.0	11.0-15.0 g/dL
Hematocrit	27.2	26.6	35-46%
White blood cell	13.6	7.4	3.8-5.3 10x6/uL
Platelets	90,000	200,000	130-400 10x3/uL
Troponin	80.6	78.8	5.0-19.7 pg/mL
Glucose	91	120	80-115 mg/dL
Blood urea nitrogen	85	39	9.8-20.1 mg/dL
Creatinine	12.2	9.1	0.57-1.11 mg/dL
Sodium	135	138	136-145 mmol/L
Potassium	5.2	3.9	3.5-5.1 mmol/L
Chloride	96	99	98-107 mmol/L
Bicarbonate	24	28	23-31 mmol/L
Calcium	8.7	8.0	8.8-10.0 mg/dL
Albumin	3.8	3.4	3.2-4.6 g/dL
Magnesium	2.3	2.2	1.6-2.6 mg/dL
COVID PCR	Negative	-	Negative
Prothrombin time	16.1	15.0	9.8-13.4 sec
international normalised ratio (INR)	1.48	1.41	0.85-1.15
Partial thromboplastin time (PTT)	30.2	33.8	24.9-35.9 sec
Thyroid-stimulating hormone	1.58	-	0.465-4.680 uIU/mL
Urine toxicology	Negative	-	Negative

**Figure 2:**
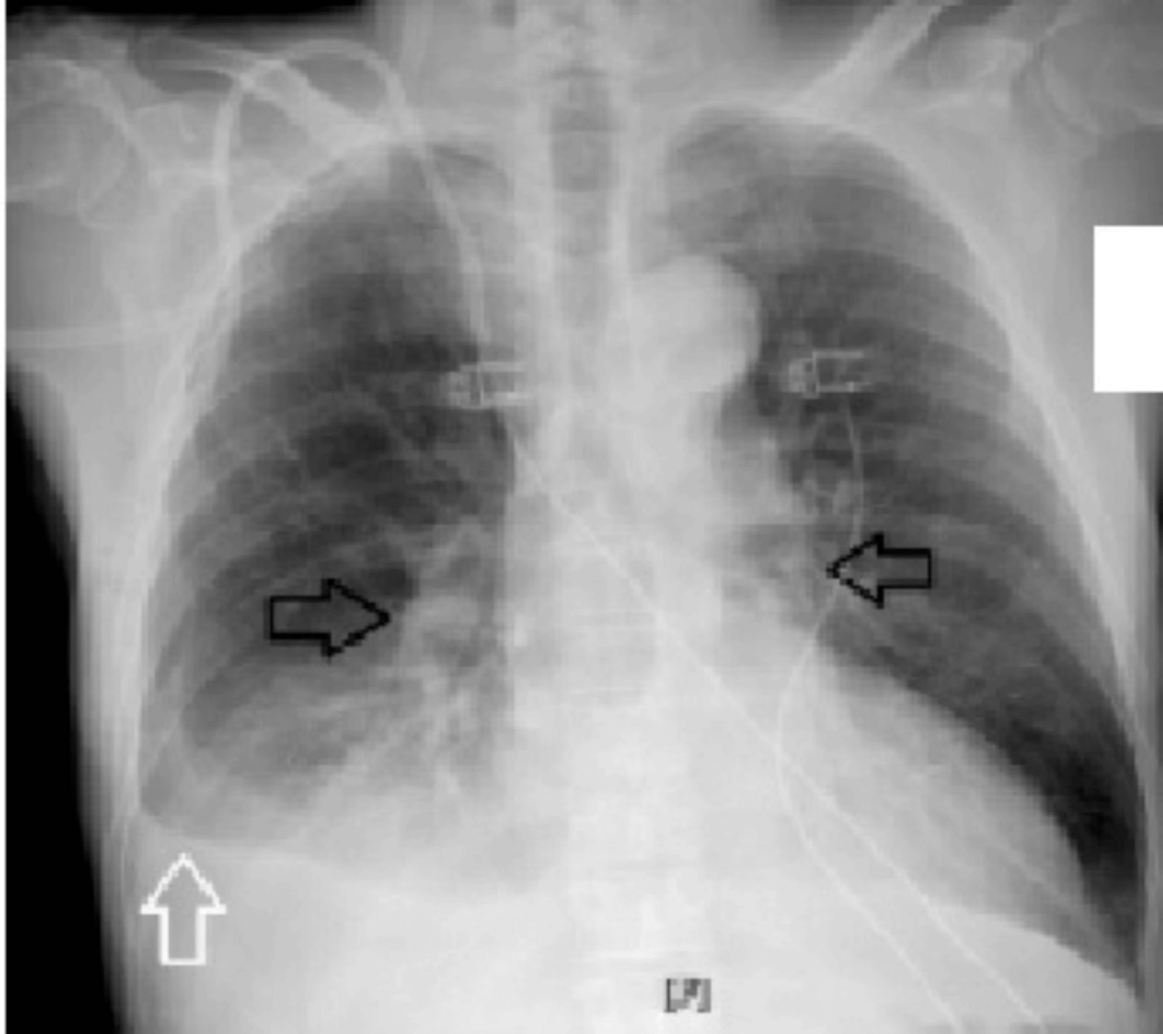
Chest X-ray showing pulmonary congestion (black arrows) and pleural effusion (white arrow).

Chest X-ray revealed pulmonary edema and pleural effusions (Figure 2).

**Figure 3:**
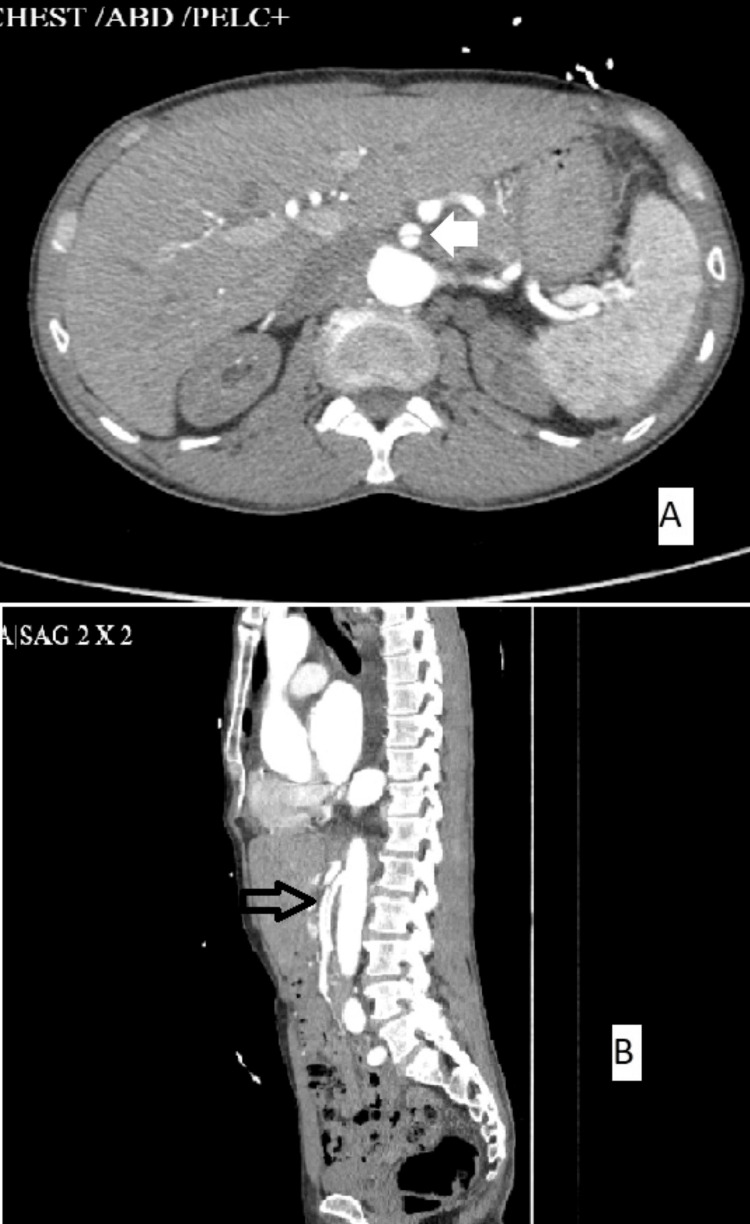
Computed tomography angiography of the abdomen showing dissection of the superior mesenteric artery (white arrow in image A and black arrow in image B).

Urgent CTA of the abdomen and pelvis was performed to exclude aortic dissection or pulmonary embolism. No aortic dissection was identified. Incidentally, an isolated dissection of the SMA was noted (Figure 3). The imaging demonstrated an intimal flap in the proximal SMA, with a patent true lumen and a false lumen extending distally, without occlusion of any branches. Bowel loops enhanced normally, and there was no evidence of pneumatosis or mesenteric venous gas.

**Figure 4:**
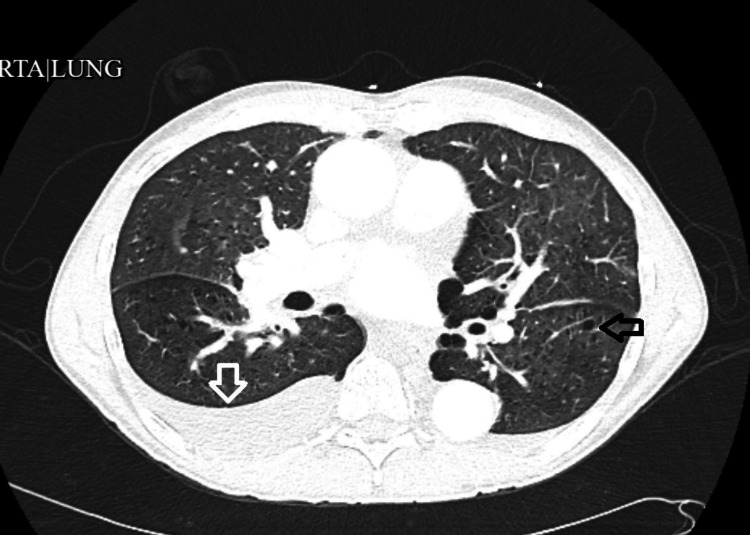
Computed tomographic angiography of the chest showing moderate emphysema (black arrow) and pleural effusion (white arrow).

CTA of the chest showed moderate emphysema and pleural effusion (Figure 4).

The vascular surgery team evaluated the patient and recommended conservative management, given the absence of abdominal symptoms or signs of ischemia, as the true lumen remained patent and bowel imaging was stable. The patient was admitted to the intensive care unit for close monitoring and management. The patient was treated for a hypertensive emergency. Intravenous antihypertensive therapy with esmolol and nicardipine infusions was initiated to achieve rapid blood pressure control, targeting less than 140/90 mmHg. Volume overload was addressed with two urgent hemodialysis sessions, removing 2-3 L of fluid via ultrafiltration. With diuresis and blood pressure control, his pulmonary edema improved, and bilevel positive airway pressure was gradually weaned by hospital day three.

Therapeutic anticoagulation was initiated for the SMA dissection. The patient initially received a heparin infusion, followed by a transition to apixaban 5 mg twice daily for outpatient therapy. Although the benefit of anticoagulation in isolated spontaneous SMA dissection remains uncertain, it was chosen to reduce the risk of thrombosis in the false lumen.

During hospitalization, the patient's abdominal examinations remained benign, with no pain or tenderness. Serial lactate levels and hemoglobin remained stable, indicating no ischemic or bleeding complications. Cardiology supervised the management of hypertensive cardiac injury and added oral antihypertensive agents - including labetalol, nifedipine, and hydralazine - as intravenous infusions were tapered. Nephrology continued management of dialysis and anemia of chronic kidney disease, maintaining erythropoiesis-stimulating agents and intravenous iron. Hematology evaluated thrombocytopenia and ruled out heparin-induced thrombocytopenia; the platelet count recovered spontaneously with supportive care. Heparin-induced thrombocytopenia and serotonin-release assay were negative. A workup for multiple myeloma was planned on an outpatient basis.

By hospital day four, the patient was breathing comfortably on a nasal cannula, and blood pressure was well controlled on oral medications. He reported no abdominal complaints. The patient was discharged home on hospital day five with instructions to continue antihypertensive therapy and apixaban (renal adjusted dosing). Follow-up was arranged with vascular surgery and primary care, with outpatient CTA or duplex ultrasound planned in three months to assess remodeling of the SMA.

## Discussion

This case illustrates several important aspects of ISMAD, particularly in the complex setting of ESRD and hypertensive emergency. Although ISMAD classically presents with acute abdominal pain, it can occasionally be clinically silent, as demonstrated in this case. Most published series report abdominal pain as the predominant symptom; however, a subset of patients may be asymptomatic or minimally symptomatic and are often diagnosed incidentally during imaging performed for unrelated indications [1,2]. Our patient's SMA dissection was identified during CTA performed for suspected vascular pathology, in the absence of any abdominal complaints, highlighting the importance of careful evaluation of imaging beyond the primary indication [1,3]. The patient's clinical profile aligns with the typical epidemiology of ISMAD, which predominantly affects middle-aged men with underlying vascular risk factors, such as hypertension and atherosclerosis [1,4]. Systemic hypertension is one of the most frequently reported comorbidities and contributes to increased arterial wall stress and intimal injury [1,5].

Additionally, the presence of hyperlipidemia and ESRD further contributes to vascular pathology through mechanisms such as accelerated atherosclerosis, vascular calcification, and chronic uremia [6,7]. While ESRD is not a well-established independent risk factor for ISMAD, it likely creates a vulnerable vascular environment that may predispose patients to arterial injury [2,3].

CTA remains the imaging modality of choice for diagnosing ISMAD due to its high sensitivity and ability to delineate the intimal flap, true and false lumens, and branch vessel perfusion [1,8]. Current literature and expert consensus recommend conservative therapy for hemodynamically stable patients without complications such as bowel ischemia, persistent pain, or progression of dissection [1,2,8].

In contrast, endovascular or surgical intervention is reserved for patients with complications, including true lumen compromise, aneurysmal dilation, or intestinal infarction [8]. A critical component of management in this case was prompt and controlled blood pressure reduction. Hypertensive emergency, particularly when associated with acute pulmonary edema, carries a high risk of morbidity and mortality and requires immediate intervention [1,7,8]. Current guidelines recommend rapid but controlled lowering of blood pressure using intravenous agents, followed by transition to oral antihypertensive therapy once stabilization is achieved [7,8]. Strict blood pressure control is also essential in ISMAD to prevent propagation or rupture of the dissection, with some literature emphasizing tighter targets in these patients [2,7]. Volume management was equally important, particularly given the patient's ESRD status. Volume overload is a major contributor to hypertension and acute heart failure in dialysis patients, and effective ultrafiltration plays a key role in achieving hemodynamic stability [5]. Close monitoring during hospitalization confirmed clinical stability, with no abdominal symptoms or bowel ischemia. Follow-up imaging is recommended for ISMAD, as conservative management often leads to stabilization or complete remodeling. Multidisciplinary care addressed comorbidities: cardiology managed demand-related myocardial injury, nephrology optimized dialysis, and transient thrombocytopenia resolved with supportive care and outpatient follow-up.

## Conclusions

Isolated SMA dissection is a rare but increasingly recognized entity due to advanced imaging. This case underscores that ISMAD can be found incidentally even in the absence of abdominal pain, especially in patients undergoing evaluation for other vascular emergencies. In hypertensive patients, especially those on dialysis, vigilant attention to blood pressure and volume is key. Uncomplicated ISMAD without bowel compromise can be managed conservatively: blood pressure reduction, possible anticoagulation, and supportive care. Vascular surgery consultation and serial exams are prudent to detect any evolution. With this approach, outcomes are excellent, and surgery is rarely needed. We recommend that clinicians consider visceral artery dissections in hypertensive crises and emphasize multidisciplinary management and follow-up in complex patients.
